# Recombinant CA Protein-Based ELISA for Serological Detection of Small Ruminant Lentiviruses Antibodies

**DOI:** 10.1155/tbed/6696495

**Published:** 2025-07-30

**Authors:** Xiaohua Ma, Yonghong Liu, Bofang Duan, Hua Gao, Qixin Huang, Cheng Chen, Ming Nie, Zhenjie Zhang, Zhihong Wu, Kui Guo, Zhe Hu, Cheng Du, Xiaojun Wang, Xue-Feng Wang

**Affiliations:** ^1^State Key Laboratory for Animal Disease Control and Prevention, Harbin Veterinary Research Institute, The Chinese Academy of Agricultural Sciences, Harbin 150069, China; ^2^College of Veterinary Medicine, Inner Mongolia Agricultural University, Hohhot 010018, China; ^3^Yunnan Center for Animal Disease Prevention and Control, Kunming 650201, China; ^4^Keerqin District Center for Animal Disease Prevention and Control, Tongliao 028000, China; ^5^Haixi Center for Animal Disease Prevention and Control, Haixi Mongolian and Tibetan Autonomous Prefecture 817099, China; ^6^Ningde Institute of Agricultural Sciences, Fu'an 355000, China; ^7^Alxa Left Banner Centre for Animal Disease Prevention and Control, Alxa 750399, China; ^8^College of Animal Science and Technology, Tarim University, Alaer 843300, China; ^9^Agriculture and Animal Husbandry Technology Popularization Center of Inner Mongolia Autonomous Region, Hohhot 010010, China; ^10^Institute of Western Agriculture, The Chinese Academy of Agricultural Sciences, Changji 831100, China

## Abstract

Maedi-visna (MV) and caprine arthritis encephalitis (CAE) are important viral diseases of sheep and goats. The diseases are caused by a group of genetically closely related lentiviruses known as small ruminant lentiviruses (SRLV), and are collectively referred to as SRLV infections. As the majority of sheep and goats infected with SRLV are asymptomatic, the disease is often overlooked. However, SRLV infection can significantly reduce animal productivity and impede animal trade. Currently, SRLV infection is widespread worldwide, but knowledge of its prevalence in China is limited due to a lack of cost-effective testing. In this study, we successfully developed an indirect enzyme-linked immunosorbent assay (iELISA) based on the SRLV capsid protein (CA) (p28) for the specific detection of anti-SRLV antibodies in serum. Using the application of checkerboard titration to optimize the iELISA assay conditions, the cut-off value was determined to be 0.09 by analyzing S/P values of 181 negative sera against SRLV that were confirmed with western blotting (WB). The method showed good reproducibility, with intra- and inter-assay coefficients of variation (CV) values less than 6.60%. A specificity test showed that the iELISA had no serological cross-reaction with six common small ruminant pathogens. Moreover, it was found to be 400–1600 times more sensitive than the available AGID test. The 93 clinical serum samples that tested SRLV-positive using the iELISA were all confirmed as positive using WB, indicating that the method has a low false positive rate. The iELISA was then applied to assess 4786 clinical serum samples from 13 cities in six provinces in China. The results show that the SRLV positivity rate of sera ranged between 0.85% and 40.00%, with the overall seroprevalence of SRLV being 10.64%. Our results indicate that the developed iELISA will serve as a valuable and efficient screening tool for the large-scale preliminary surveillance and monitoring of SRLV infections in both sheep and goats.

## 1. Introduction

Maedi-visna virus (MVV) and caprine arthritis encephalitis virus (CAEV) are members of the genus Lentivirus in the family *Retroviridae*. The two viruses were originally isolated from sheep and goats, respectively, and it was concluded that MVV only infects sheep, where the disease is called maedi-visna (MV) and CAEV only infects goats, where the disease is called caprine arthritis encephalitis (CAE) [1, 2]. For a long time, the viruses were treated as two different, strictly host-specific viruses, and the World Organization for Animal Health (WOAH) included MV and CAE in its “Listed Diseases” of terrestrial and aquatic animals. Subsequent studies have, however, shown that both viruses can be transmitted between sheep and goats and can also infect some wild ruminants [3–7]. Therefore, an increasing number of studies have collectively referred to these two viruses as small ruminant lentiviruses (SRLV), and to the disease they cause as SRLV infection [8]. Clinical and subclinical SLRV infections are often associated with progressive inflammatory lesions in the lungs, joints, mammary glands, and central nervous system. Although the majority of animals infected with SRLV do not show typical clinical signs, the disease always causes significant economic losses by reducing milk production and shortening both duration of lactation and length of life [9–11].

Like other lentiviruses, the SRLV genome is highly diverse, with relatively conserved *gag* and *pol* genes and highly variable *env* genes. Phylogenetic analyses of the *gag-pol* sequence showed that SRLV can be classified into 5 genotypes, A–E, with more than 30 subtypes [12, 13]. The lentiviral structural proteins Gag and Env are major targets for the humoral immune response and are primary targets for serologic diagnosis. The capsid protein (CA), which is cleaved from the Gag precursor protein, is highly conserved and is the most abundant protein in the lentiviral virions. This protein is the first antigen recognized by infected animals, and antibodies to it are detected long after infection. Therefore, CA has been widely used as a serological test for lentiviral diagnosis [14–16].

SRLV infections are widespread worldwide and pose a serious risk to the production and international trade of small ruminants (sheep and goats) [17–25]. Neither a vaccine against the disease nor treatment for SRLV infection is available to date, and early detection of SRLV-positive animals using appropriate diagnostic tests and prevention of virus transmission in the herd is the only effective strategy for prevention and control of the disease. However, epidemiological data on the disease in China are limited due to the lack of a commercially available, low-cost diagnostic method, which limits the proposal and implementation of targeted national control programs.

Enzyme-linked immunosorbent assay (ELISA) is known for its relatively low-cost and high throughput, advantages that make it the method of choice for detection and large-scale surveillance of many infectious diseases. In this study, we developed an indirect ELISA (iELISA) based on the SRLV CA protein (p28) for the specific detection of antibodies against SRLV in serum. The prevalence of SRLV in some cities in China was subsequently investigated using this method.

## 2. Materials and Methods

### 2.1. Development and Optimization of an iELISA Based on p28

A recombinant p28 protein with a His tag was derived from the SRLV-X strain (GenBank accession number PP854830), which belongs to the A2 genotype of SRLV [26]. This protein was used as the coating antigen in this study. The experimental conditions for the iELISA were optimized using a checkerboard assay. The ratio of positive to negative serum OD_450_ values (P/N) was calculated, and the conditions with the highest P/N ratio were used to determine the following experimental conditions: optimal antigen coating concentration (p28 protein was coated separately at 100, 150, 200 and 300 ng/well), primary antibody dilution (SRLV-positive and negative serum were diluted at 1:100, 1:200, 1:300 and 1:400), secondary antibody dilution (monoclonal anti-goat/sheep IgG–peroxidase antibody produced in mouse, Sigma–Aldrich, USA, was diluted at 1:10,000, 1:20,000, and 1:40,000), blocking buffer and reaction time (5% skim milk, 1% BSA, and 1% gelatin, placed at 37°C for 30, 60, 90, and 120 min, respectively), and substrate solution (TMB; Beyotime, China) reaction time (5, 10, 15, or 20 min at room temperature). The optimal duration of the serum (37°C for 60, 90, 120, or 150 min) and secondary antibodies (37°C for 15, 30, 45, or 60 min) were then identified from the conditions determined above.

The mean value (M) and standard deviation (SD) of S/P values from 181 SRLV-negative serum samples were used to estimate the cut-off value, and 34 SRLV-positive samples were used as controls. S/P values above M + 3SD were considered positive. The S/P value was calculated as follows:

  $$\begin{array}{c} \text{S}/\text{P value}=(\text{sample value}-\text{ negative control value})/(\text{positive control value}-\text{ negative control value}). \end{array}$$

### 2.2. Evaluation of the Specificity and Reproducibility of the iELISA

The antisera against peste des petits ruminants virus (PPRV), *B. melitensis*, foot-and-mouth disease virus serotype A (FMDV-A), foot-and-mouth disease virus serotype O (FMDV-O), sheep pox virus (SPPV), and Orf virus (ORFV) were tested to validate the specificity of the iELISA. These serum samples were kindly provided by Weiye Chen (Harbin Veterinary Research Institute, Chinese Academy of Agricultural Sciences, for antisera against PPRV and *B. melitensis*), Mingqi Dong (National Engineering Research Center of Veterinary Biologics, China, for antisera against FMDV-A, FMDV-O and SPPV), and Zhizhong Jing (Lanzhou Veterinary Research Institute, Chinese Academy of Agricultural Sciences, for antisera against ORFV).

To evaluate the intra-assay reproducibility and inter-assay repeatability, SRLV-positive serum samples at different dilutions (400×, high; 800×, medium; and 1600×, low) and SRLV-negative serum samples were tested in three replicates or three independent iELISA plates, respectively.

### 2.3. Comparison of iELISA With Western Blotting (WB) and AGID Test

To compare the sensitivity of iELISA and AGID, 93 clinical serum samples that tested SRLV-positive using the iELISA were diluted 1:50 as primary antibody, and then WB assays were performed using purified p28 as test antigen and IRDye 800CW donkey anti-goat IgG secondary antibody (diluted 1:5000, LI-COR Biosciences, Lincoln, NE USA) as secondary antibody, as previously described [26]. All of these serum samples were subjected to a twofold dilution, after which an AGID test was performed according to the manufacturer's instructions (Maedi-Visna Agar Gel Immunodiffusion Assay Kit, RAI0259, APHA Scientific, United Kingdom).

### 2.4. Comparison of iELISA With a Commercial Kit

To evaluate the diagnostic performance of the developed iELISA, 159 clinical serum samples were tested in parallel using both the iELISA and a commercial ELISA kit following the manufacturer's instructions (ID. Vet, CVT1135T).

### 2.5. Detection of SRLV Antibodies in Field Serum Samples Using the iELISA

A total of 4786 test serum samples were collected from 13 cities in China (Ordos from 2019 to 2021; Chifeng in 2022; Hohhot from 2023 to 2024; Tongliao in 2024; Alxa in 2024; Daqing in 2023; Kunming in 2024; Ningde in 2024; Haixi Mongolian and Tibetan Autonomous Prefecture in 2024; Kashgar in 2024; Ili Kazakh Autonomous Prefecture in 2024; Turpan in 2024 and Bayingolin Mongolian Autonomous Prefecture in 2024) and tested using the established iELISA method described above.

## 3. Results and Discussion

**Figure 1:**
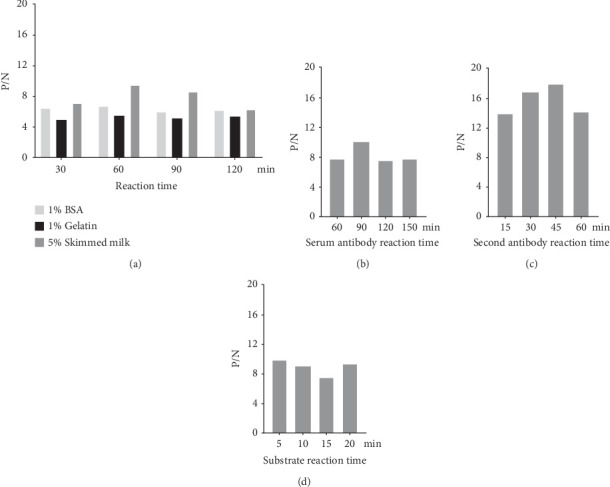
Experiments assessing the optimal conditions for the iELISA. (A) Optimal blocking solution and reaction time. (B) Optimal reaction time for serum. (C) Optimal reaction time of secondary antibody. (D) Optimal substrate–enzyme interaction time.

To develop a p28-based iELISA for the detection of SRLV infection, a recombinant p28 protein with His tag was used as the coated antigen and the optimal working-conditions (with the highest P/N ratio value) were determined using checkerboard titrations as follows. As shown in Table 1, the optimal coating antigen concentration was determined to be 300 ng/well, the optimal serum dilution as primary antibody was found to be 1:400, and the optimal secondary antibody dilution was 1:10,000. For blocking conditions, we found using 5% skim milk for 60 min exhibited the highest blocking effect (Figure 1A). The optimal reaction time for the primary antibody was 90 min (Figure 1B), the optimal reaction time for secondary antibody was 45 min (Figure 1C), and the substrate-enzyme reaction time was 5 min (Figure 1D).

**Figure 2:**
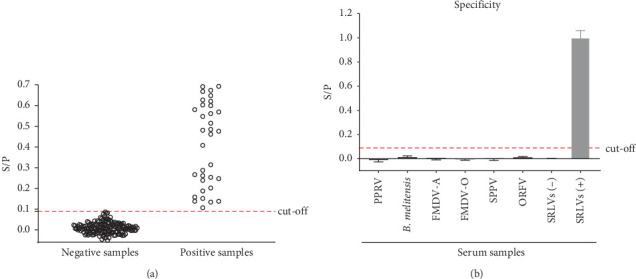
Evaluation and application of the established iELISA method. (A) Cut-off values were determined using 181 clinically negative serum samples, with 34 positive samples were used as positive controls. (B) The specificity of the iELISA was evaluated for antisera against common sheep and goat pathogens including PPRV, *B. melitensis*, FMDV-A, FMDV-O, SPPV, and ORFV.

A total of 181 WB-verified SRLV-seronegative samples were evaluated using the established iELISA method and 34 WB-verified SRLV-seropositive samples were used as positive controls. As shown in Figure 2A, the mean S/P value (M) of these sera was 0.0126 with an SD of 0.0283. Therefore, the cut-off value for the iELISA was defined as 0.09 (M + 3SD). In other words, all serum samples with S/P values at or above this cut-off value were considered positive, and serum samples with S/P values less than 0.09 were assessed as negative.

A panel of antisera against PPRV, *B. melitensis*, FMDV-A, FMDV-O, SPPV and ORFV was used to evaluate the specificity of the iELISA. As shown in Figure 2B, the S/P values for all of the above serum samples were less than 0.09, indicating that the established iELISA did not cross-react with antisera against any of the above pathogens. The reproducibility of the iELISA was evaluated using SRLV-positive sera at different dilutions (400×, high; 800×, medium; 1600×, low) and a SRLV-negative serum sample in three replicates in three independent ELISA assays. As shown in Table 2, intra- and inter-assay coefficients of variations (CVs) of the iELISA were found to be 2.76%–4.37% and 5.13%–6.60%, respectively.

The AGID test and ELISA are the methods recommended by the WOAH for the diagnosis of SRLV [27]. Here, we compared the sensitivity of the iELISA developed in this study and a commercially available AGID kit. Four SRLV-positive serum samples verified with WB were tested using both the established iELISA and a commercial AGID kit (targeting gp135). As shown in Table 3, the maximum dilutions at which these serum samples tested positive using the iELISA ranged between 400× and 1600×, whereas only two undiluted samples tested positive with the AGID. Following the recommendations of the WOAH Terrestrial Animal Code [27], we then compared the iELISA results with those from WB, and found that the 93 clinical serum samples that tested positive using the iELISA were also all found to be positive when tested using WB (Figure S1), indicating that the iELISA has a low false positive rate. Subsequently, 93 field serum samples that tested for SRLV using both iELISA and WB were analyzed using the AGID method. Of these 93 samples, only 16 tested positive using AGID (Table 4). The results highlight that the iELISA established with recombinant p28 demonstrates remarkable performance, exhibiting a sensitivity 400–1600 times higher than the AGID test targeting gp135. Therefore, the iELISA developed in this study is also suitable for screening SRLV carriers in the context of the uncertain genomic characteristics of circulating SRLV strains in China.

Furthermore, we performed a comparative analysis of the p28-based iELISA and a commercial ELISA kit (ID.Vet, CVT1135T), the latter of which uses a combination of synthetic peptides derived from p28, gp135 and gp45 [28]. Analysis of 159 clinical serum samples revealed positive rates of 11.32% (18/159) and 33.33% (53/159) using the iELISA and the commercial ELISA kit, respectively. The agreement rate between the iELISA and the commercial ELISA kit was 76.73% (122/159) (Table 5). All 18 samples that tested positive using the iELISA also tested positive using WB (data not shown). Howere, the 17 of 18 samples also tested positive using the commercial kit. This result suggests that the iELISA may produce false negatives, but not false-positive. PCR detection could potentially exclude false-negative results [29]. However, the limited volume of serum samples collected in this study and the absence of corresponding blood or tissue samples did not allow us to perform PCR analysis. Previous studies have demonstrated that antibodies against p28 can be detected early during SRLV infection and persist for extended periods [30]. In contrast, antibodies targeting Env protein typically appear in later stages during SRLV infection and may reach higher titers in some individuals [31, 32]. Combining p28 with a fraction of Env (gp135) increases the sensitivity of diagnostic tests for SRLV antibodies compared to using p28 alone [33]. We therefore attempted to express p28 fuzed with a portion of Env, as in a previous study [34], but were unable to obtain the recombinant protein. Thus, the use of a single antigen p28 may be responsible for the lower positive rates observed in the established iELISA compared to the commercial kit. Nevertheless, we cannot rule out the possibility of false-positive results from this commercial kit. A WB analysis using whole virus could be a reliable method of identifying false positives [35]. Unfortunately, our laboratory currently lacks the capability to culture SRLV, therefore whole-virus WB detection cannot be performed.

Next, the iELISA method established in this study was used to detect 4786 serum samples collected from 13 cities in six provinces in China between 2019 and 2024. As shown in Table 6, the rate of positivity of SRLV-specific antibodies ranged from 0.85% to 40.00% in the different cities. To date, there are few reports on the prevalence of SRLV infection in China using imported commercial kits. A seroprevalence study of SRLV by Sun et al. [36] in 2018 showed an overall mean positivity rate of 0.41% (*n* = 3422) from 11 regions in China, with 0 positivity (from 297 samples) in Inner Mongolia Autonomous Region and Qinhai Province (from 278 samples), whereas in the present study, positivity rates in Inner Mongolia ranged between 0.85% and 17.15%, with a mean positivity rate of 10.40% (369/3547); the positivity rate in Haixi Mongolian and Tibetan Autonomous Prefecture, Qinghai Province is 7.4% (34/460). A study more than a decade ago showed that the seroprevalence of SRLV in sheep in 2011 was 4.6%–50% in 12 regions in China, mainly concentrated in the southern and northwestern regions of China [37]. A study of SRLV serological surveys in sheep flocks in certain areas of Xinjiang showed that SRLV seroprevalence in the region ranged from 0.67% to 28.95% in 2017–2019 [38], whereas the SRLV positivity rate in Xinjiang in the present study ranged from 2.86% to 40.00%, with a mean positivity rate of 18.87% (57/302). Here we found that the seroprevalence of SRLV was 21% (*n* = 100) on a sheep farm in Daqing city, Heilongjiang Province, northeast China. More recent research demonstrated that the molecular prevalence of SRLV in Jiangsu and Shanghai was 0.77% (*n* = 780) [39]. In the present study, we found that the seropositivity of SRLV in a city in southern China, Ningde City, Fujian Province, was 9.54% (*n* = 262). Although these data show that there are differences in rates of SRLV positivity in different regions of China, the possibility of sampling bias cannot be ruled out. The high genetic heterogeneity of the virus and the low viral load of infected animals may also hinder the effectiveness of SRLV diagnosis. Furthermore, most current studies rely on a single diagnostic test, which may underestimate the true prevalence of infection in China. Notably, the present study shows that animals raised under intensive farming conditions (11.76%, 140/1190; from Chifeng, Hohhot, Tongliao, Daqing, Kashgar, and Bayingolin Mongolian Autonomous Prefecture) had a significantly higher seroprevalence rate than animals raised under grazing conditions (6.24%, 44/705; from Alxa, Kunming, Haixi, Kashgar, and Turpan). This difference may be due to environmental and management factors inherent in intensive production systems, such as increased animal density, frequent close contact, and increased physiological stress, all of which can enhance the efficiency of virus transmission. Overall, however, the above data suggest that SRLV infection is widespread in China.

It is well known that SRLV exhibit a high degree of genetic and antigenic variability, which poses severe challenge to the diagnostic of SRLV infections. Studies have shown that the prevalent strains of SRLV in China during the 1980s and 1990s were subtypes A2 and B1 [40, 41]. However, there has been a lack of reports on the SRLV strains circulating in recent years. Thus, the iELISA established in this study using the p28 from the subtype A2 SRLV may miss animals infected with different genotypes [42, 43]. In this study, detection results from several distinct flocks of goats (from Huhhot, Kunming and Ningde) and sheep (from Chifeng, Tongliao, and Daqing) showed that the SRLV positive rate was 9.84% (54/549) in sheep and 7.57% (64/846) in goat. Therefore, it is unable to determine whether the established iELISA has different diagnostic efficiency for SRLV subtypes. Given the poor availability of epidemiological data on the SRLV infection in China, it is necessary to perform nucleic acid testing (PCR) in follow-up studies and conduct a systematic phylogenetic analysis of the SRLV strains circulating in China to clarify their subtype information. Our aim is to further optimize the serological and molecular methods for SRLV infection based on the characteristics of prevalent SRLV strains in China, so as to improve the diagnostic precision. Importantly, this study provides preliminary data on the epidemiology of SRLV in China, and highlights the need for continuous surveillance of SRLV prevalent in different regions, which may help to modify and develop more appropriate strategies and methods for the prevention and control of SRLV infection in China. Future studies should focus on elucidating SRLV transmission dynamics within populations and different farms, as well as identifying risk factors associated with infection. These efforts will help improve current prevention strategies and develop new diagnostic and control methods for the epidemiological situation of SRLV in China.

## 4. Conclusion

We have successfully established a p28-based iELISA and demonstrated that this method can be used for large-scale screening of antibodies against SRLV in clinical serum samples from sheep and goats. We also demonstrated the widespread prevalence of SRLV in China.

## Figures and Tables

**Table 1:** Optimization of concentration of coating antigen, serum dilution, and secondary antibody dilution.

Antigen (ng/well)	Secondary antibody dilution	Serum dilution											
		1:100			1:200			1:300			1:400		
		P	N	P/N	P	N	P/N	P	N	P/N	P	N	P/N
100	1:10,000	0.708	0.405	1.748	0.55	0.305	1.803	0.504	0.218	2.312	0.511	0.165	3.097
	1:20,000	0.417	0.202	2.064	0.375	0.228	1.645	0.316	0.153	2.065	0.336	0.114	2.947
	1:40,000	0.267	0.156	1.712	0.199	0.112	1.777	0.19	0.095	2.000	0.209	0.093	2.247

150	1:10,000	1.004	0.385	2.608	0.745	0.271	2.749	0.572	0.221	2.588	0.642	0.171	3.754
	1:20,000	0.506	0.259	1.954	0.427	0.162	2.636	0.381	0.139	2.741	0.402	0.115	3.496
	1:40,000	0.305	0.148	2.061	0.293	0.105	2.790	0.241	0.095	2.537	0.246	0.08	3.075

200	1:10,000	1.215	0.375	3.24	1.19	0.227	5.242	0.945	0.2	4.725	0.989	0.143	6.916
	1:20,000	0.744	0.208	3.577	0.68	0.152	4.474	0.623	0.129	4.829	0.636	0.125	5.088
	1:40,000	0.435	0.135	3.222	0.4	0.113	3.540	0.39	0.09	4.333	0.369	0.094	3.926

300	1:10,000	1.547	0.349	4.433	1.573	0.255	6.169	1.542	0.188	8.202	1.583	0.188	**8.420**
	1:20,000	0.941	0.226	4.164	0.979	0.144	6.799	0.968	0.127	7.622	0.929	0.136	6.831
	1:40,000	0.621	0.186	3.339	0.561	0.121	4.636	0.532	0.115	4.626	0.502	0.083	6.048

*Note*: The bold value indicates the maximum P/N ratio in this study.

**Table 2:** Evaluation of iELISA reproducibility.

Reproducibility	Serum sample	M ± SD	CV (%)
Inter	High (400×)	0.985 ± 0.050475	5.13
	Medium (800×)	0.804 ± 0.050475	6.57
	Low (1600×)	0.421 ± 0.023313	5.54
	Negative	0.072 ± 0.004717	6.60

Intra	High (400×)	0.985 ± 0.043079	4.37
	Medium (800×)	0.790 ± 0.023043	2.92
	Low (1600×)	0.571 ± 0.015786	2.76
	Negative	0.070 ± 0.003033	4.33

**Table 3:** Comparison of the sensitivities of the iELISA and AGID tests.

Samples		1	2	3	4
iELISA	200×	+	+	+	+
	400×	+	+	+	+
	800×	−	+	+	+
	1600×	−	+	−	−
	3200×	−	−	−	−

AGID	Undiluted	−	+	+	−
	2×	−	−	−	−

**Table 4:** Comparison of agreement between AGID and iELISA in clinical samples.

AGID	iELISA positive	iELISA negative	Total
AGID positive	16	0	16
AGID negative	77	0	77
Total	93	0	93

**Table 5:** Comparison of agreement between commercial ELISA kit and iELISA in clinical samples.

Commercial ELISA kit	iELISA positive	iELISA negative	Total
Commercial ELISA kit positive	17	36	53
Commercial ELISA kit negative	1	105	106
Total	18	141	159

**Table 6:** Seroprevalence of SRLV infections in 13 cities of China.

Area	Year	Positive rate	Breeds	Husbandry	Species
Ordos, Inner Mongolia	2019	8.95% (83/927)	⁣^*a*^	⁣^*a*^	Sheep and goat
	2020	6.25% (25/400)	⁣^*a*^	⁣^*a*^	—
	2021	8.80% (33/375)	⁣^*a*^	⁣^*a*^	—

Chifeng, Inner Mongolia	2022	11.37% (29/255)	⁣^*a*^	Intensive	Sheep

Hohhot, Inner Mongolia	2023	9.94% (35/352)	Saanen goat	Intensive	Goat
	2024	0.85% (1/117)	—	—	—

Tongliao, Inner Mongolia	2024	2.06% (4/194)	Small-tailed Han sheep	Intensive	Sheep

Alxa, Inner Mongolia	2024	17.15% (159/927)	⁣^*a*^	Free-range	Sheep and goat

Daqing, Heilongjiang	2023	21.00% (21/100)	Merino sheep	Intensive	Sheep

Kunming, Yunnan	2024	2.61% (3/115)	⁣^*a*^	Grazing with supplementary feeding	Goat

Haixi, Qinghai	2024	7.40% (34/460)	^b^Tibetan sheep and semi-fine wool sheep	Grazing with supplementary feeding	Sheep and goat

Ningde, Fujian	2024	9.54% (25/262)	⁣^*a*^	⁣^*a*^	Goat

Kashgar, Xinjiang	2024	2.86% (2/70)	^b^Daolang sheep	Free-range	Sheep and goat

Ili Kazakh Autonomous Prefecture, Xinjiang	2024	23.21% (26/112)	^b^Dorper sheep and Hu sheep	Intensive	Sheep and goat

Turpan, Xinjiang	2024	8.33% (5/60)	^b^Kazakh sheep	Free-range	Sheep and goat

Bayingolin Mongolian Autonomous Prefecture, Xinjiang	2024	40.00% (24/60)	^b^Dorper sheep and Hu sheep	Intensive	Sheep and goat

Total	—	10.64% (509/4786)	—	—	—

⁣^*a*^The informations was not documented.

^b^The breeds representing the majority of animals are displayed.

## Data Availability

The data that support the findings of this study are available in the supporting information of this article.

## Supporting Information

Supporting Information Figure S1. Determination of 93 clinical serum samples positive for SRLV by iELISA using WB, as described in Section 2. The 93 clinical samples were collected from four cities: Chifeng, Inner Mongolia (*n* = 18), Daqing, Heilongjiang (*n* = 16), Ningde, Fujian (*n* = 25), and Haixi, Qinghai (*n* = 34). The numbers below the WB images represent the sample identifiers, PC represents the positive control, and NC represents the negative control.
