# Supplementary material for: Recombinant CA Protein-Based ELISA for Serological Detection of Small Ruminant Lentiviruses Antibodies
Source: Transbound Emerg Dis. 2025 Jul 30;2025:6696495. doi: 10.1155/tbed/6696495 (PMC12328048; doi:10.1155/tbed/6696495)
Supplement: Supporting Information — Figure S1. Determination of 93 clinical serum samples positive for SRLV by iELISA using WB, as described in Section 2. The 93 clinical samples were collected from four cities: Chifeng, Inner Mongolia (n = 18), Daqing, Heilongjiang (n = 16), Ningde, Fujian (n = 25), and Haixi, Qinghai (n = 34). The numbers below the WB images represent the sample identifiers, PC represents the positive control, and NC represents the negative control. [file 6696495.f1.docx]

**“Recombinant CA Protein-Based ELISA for Serological Detection of Small Ruminant Lentiviruses Antibodies”**

Xiaohua Ma^1^, Yonghong Liu^2^, Bofang Duan^3^, Hua Gao^4^, Qixin Huang^5^, Cheng Chen^6^, Ming Nie^7^, Zhenjie Zhang^8^, Zhihong Wu^9^, Kui Guo^1^, Zhe Hu^1^, Cheng Du^1^, Xiaojun Wang^1,10^, Xue-Feng Wang^1^


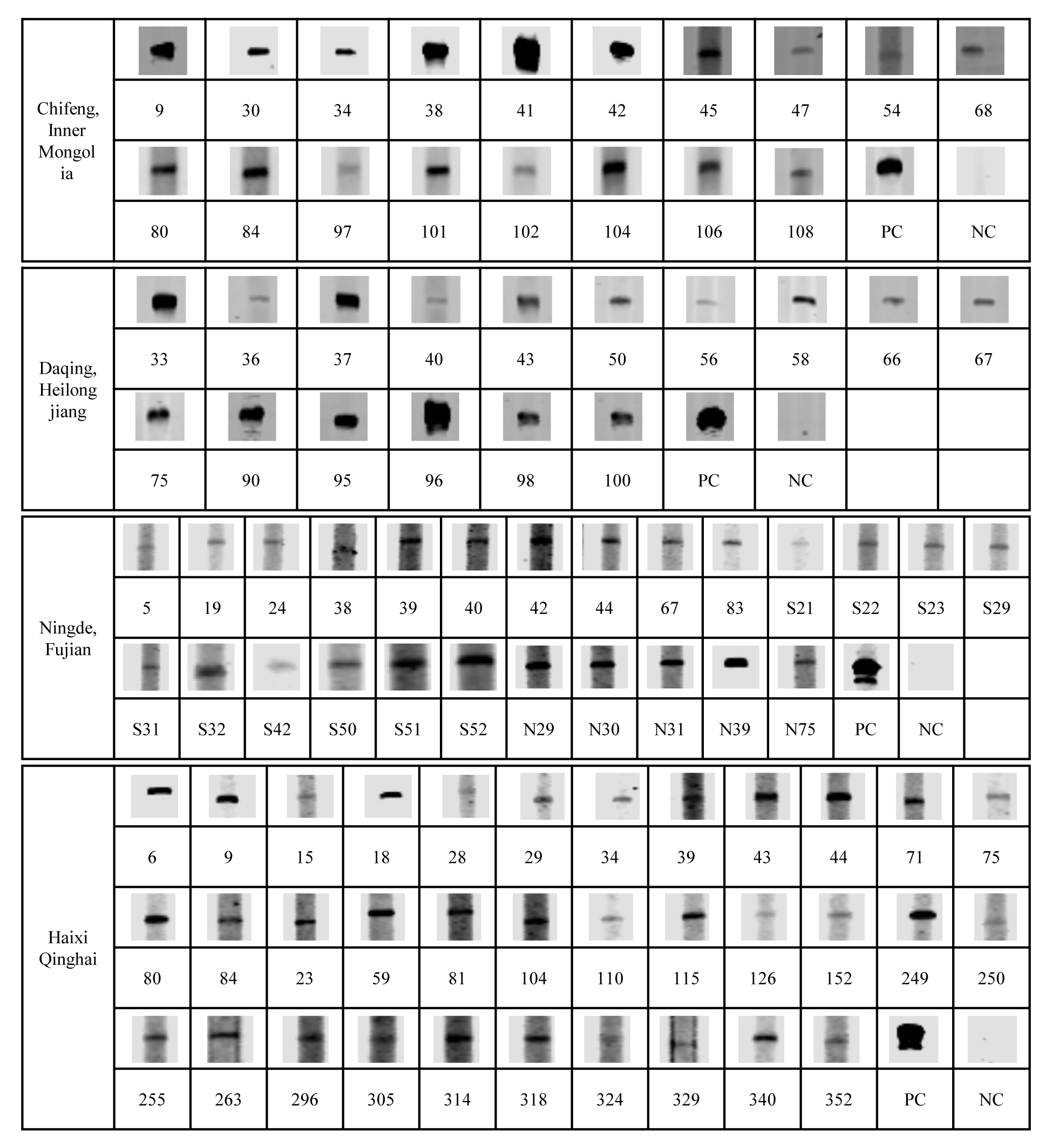
**Supplementary materials**

Figure S1. Determination of 93 clinical serum samples positive for SRLV by iELISA using WB as described in Materials and Methods. The 93 clinical samples were collected from four cities: Chifeng, Inner Mongolia (n=18); Daqing, Heilongjiang (n=16); Ningde, Fujian (n=25); and Haixi, Qinghai (n=34). The numbers below the WB images represent the sample identifiers, PC represent the positive control and NC represent the negative control.
